# Super strong wide TM Mie bandgaps tolerating disorders

**DOI:** 10.1038/s41598-022-11610-0

**Published:** 2022-05-12

**Authors:** Kiyanoush Goudarzi, Moonjoo Lee

**Affiliations:** Department of Electrical Engineering, Pohang University of Science and Technology (POSTECH), Pohang, 37673 Korea

**Keywords:** Optics and photonics, Integrated optics

## Abstract

This study demonstrates the appearance of super intense and wide Mie bandgaps in metamaterials composed of tellurium, germanium, and silicon rods in air that tolerate some disordering of rod position and rod radius under transverse magnetic (TM) polarized light waves. Tellurium metamaterials reveal ${\mathrm{TM}}_{01}$, ${\mathrm{TM}}_{11}$, ${\mathrm{TM}}_{21}$, ${\mathrm{TM}}_{02}$, ${\mathrm{TM}}_{12}$ Mie bandgap modes in which ${\mathrm{TM}}_{01}$, ${\mathrm{TM}}_{11}$, and ${\mathrm{TM}}_{21}$ tolerate high rod-position disordering of $50\%$ and rod-radius disordering of 34 and $27\%$, respectively. Results for germanium metamaterials show Mie bandgap modes ${\mathrm{TM}}_{01}$, ${\mathrm{TM}}_{11}$, and ${\mathrm{TM}}_{21}$, in which ${\mathrm{TM}}_{01}$ and ${\mathrm{TM}}_{11}$ tolerate rod-position disordering of $50\%$, and rod-radius disordering of 34 and $20\%$, respectively. Using these characteristics of ${\mathrm{TM}}_{11}$ in germanium metamaterials under position and radius disordering, ultra-narrow straight, L-shaped, and crossing waveguides that contain 14, four, and two rows of germanium rods in air are designed. Also, it is shown that ${\mathrm{TE}}_{01}$ Mie bandgap appears in metamaterials containing a high refractive index, and disappears in metamaterials with a lower refractive index such as silicon; in contrast, a new phenomenon of intense and broadband ${\mathrm{TM}}_{01}$, ${\mathrm{TM}}_{11}$, and ${\mathrm{TM}}_{21}$ in metamaterials with a lower refractive index such as silicon appear. In silicon-based metamaterials, ${\mathrm{TM}}_{01}$ tolerates high rod-position and rod-radius disordering of $40\%$ and $34\%$, respectively, and ${\mathrm{TM}}_{11}$ shows robustness to rod-position and rod-radius disordering of $20\%$. This strong tolerance of disordering of TM modes in tellurium, germanium, and silicon metamaterials opens a new way to design small, high-efficient, and feasible fabrication optical devices for optical integrated circuits.

## Introduction

Manipulation of electromagnetic waves at the scale of subwavelength structures requires strong light-matter interactions. The light-matter interaction happens in several structures such as plasmonic, photonic crystals (PCs), and all-dielectric metamaterials (MMs). The interaction in plasmonic structures is provided by the coupling of incident light to plasmons^1–4^. Plasmonic structures are composed of dissipative materials, so the interaction causes power dissipation^5–9^. Other structures such as PCs and all-dielectric MMs show strong light-matter interactions and are feasible candidates to overcoming this power-dissipation problem^10–13^. The phenomenon of light-matter interaction in PCs appears in the form of the Bragg effect^14^, which has its origin in the periodic nature, and generates Bragg bandgaps in the photonic band structures. The produced bandgaps act as mirrors that prohibit propagation of incident light through the PCs. Point, line, and planar defects inside the PCs can localize guided modes in the bandgaps; this phenomenon provides an opportunity to steer light inside the PCs. Although PCs are suitable structures for designing low-loss and high-efficient optical devices, they manipulate light at the scale of a wavelength, and therefore must be large^14–16^.

The best replacement structures to design of small optical devices that have low dissipation are all-dielectric MMs^12,13^. These are artificial dielectric structures in the form of periodic arrays in which each unit cell contributes to the functionality of the structure. The occurrence of Mie resonances, i.e., scattering of light by small particles, in all-dielectric MMs provides a new way to steer light through the optical devices^17–20^. The high refractive index *n* of elements in all-dielectric MMs permit small size, and their dielectric natures permit ultra-low power consumption. High-*n* elements manipulate incident light in a fraction $\frac{\lambda }{n}$ of wavelength $\lambda$. The dielectric nature of these elements results in low power dissipation in all-dielectric MMs.

As feature size is scaled down in subwavelength structures, fabrication imperfections become increasingly significant, because they impose disordering that can eliminate some of the structures’ functionalities^21–28^. The disordering up to a certain level are eliminated using all-dielectric MMs that are composed of elements that have high *n* under TE polarized light waves. The transition from photonic crystals that have low-*n* elements ($n = 2$) to all-dielectric MMs that have high-*n* elements ($n = 5$) yields a ${\mathrm{TM}}_{01}$ Mie bandgap that is insensitive to position disordering of 40%^29^. The transition from PCs to all-dielectric MMs can be achieved in two ways^30^. The first is to increase *n* of the elements, while maintaining a constant ratio of radius *r* of the elements to the period *a* of the structure. The second is to increase $\frac{r}{a}$ while maintaining *n*. The goal of both ways is to increase the effective refractive index $n_{\mathrm{eff}}$ of the structure, and thereby yield Mie bandgaps in the dispersion diagram and transmission spectra^30^.

Proposing optical integrated circuits (OIC) in 1969 by S. E. Miller has attracted many researchers’ attentions to design on-chip optical components. The on-chip OICs require small and highly-efficient optical components which are compatible with CMOS fabrication technology. The optical components such as waveguides^31,32^, power splitters^33–35^, demultiplexers^36–38^, and crossing waveguides^39,40^ are mainly used for steering, filtering, and splitting light waves. Among the optical components, straight, L-shaped, and crossing waveguides are vital for OICs. High-*n* all-dielectric MMs-based waveguides are low-loss and shrank components for OICs. The rapid advancement of high-*n* all-dielectric MMs in photonics is accompanied by the inevitable cost of fabrication imperfections. In high-*n* all-dielectric MMs that contain dielectric rods in air, these imperfections appear as position and radius disordering, as a result, designing straight, L-shaped, and crossing waveguides which tolerate fabrication imperfections are of great interests for OICs.

This paper for the first time of our best knowledge presents a study of the tolerance of ${\mathrm{TM}}_{01}$, ${\mathrm{TM}}_{11}$, and ${\mathrm{TM}}_{21}$ Mie bandgaps to the rod-position and rod-radius disordering in all-dielectric MMs, then proposes and evaluates ultra-narrow straight, L-shaped, and crossing waveguides composed of two or four rows of germanium (Ge) rods. Ge has a high-*n* of about 4 over the wavelength range of 2 $\upmu$m $< \lambda<$ 11 $\upmu$m^41^. The proposed all-dielectric MMs use Ge rods in a cubic arrangement in air. Also, a comparison between TE and TM Mie bandgaps in tellurium (Te), Ge, and silicon (Si) MMs with high-*n* to lower *n*, respectively, is presented. The comparison shows existence of intense, robust to disordering and broadband ${\mathrm{TM}}_{01}$, ${\mathrm{TM}}_{11}$, and ${\mathrm{TM}}_{21}$ Mie bandgaps and diminishing ${\mathrm{TM}}_{01}$ in the MMs. The structures were simulated using the Maxwell’s equations solver of the two-dimensional finite-difference time-domain (FDTD) numerical method imbedded in the FDTD module of Lumerical software.

## Physical background

Mie and Fabry-Perot (FP) resonances govern the physical background of high-*n* all-dielectric MMs. Shining a plane wave on a high-*n* dielectric rod that has length *L* $\gg$ radius *r* causes $a_l^{m}$ and $b_l^{m}$ resonances (where *m* and *l* are integer mode numbers), which are caused by strong light-matter interactions. Transverse magnetic (TM) polarized illumination yields $a_l^{m}$, and transverse electric (TE) polarized illumination yields $b_l^{m}$. A transverse wavevector $k_{\bot }^{l}= \frac{2\pi }{\lambda _{l}}$ (where $\lambda _{l}$ is the Mie resonant wavelength for different *l* mode number) is created that is responsible for Mie resonances, and a longitudinal wavevector $k_{\Vert }^{m}= \frac{m\pi }{L}$ is created that is responsible for the FP resonance appears as a result of the length of the rod^42^.

**Figure 1:**
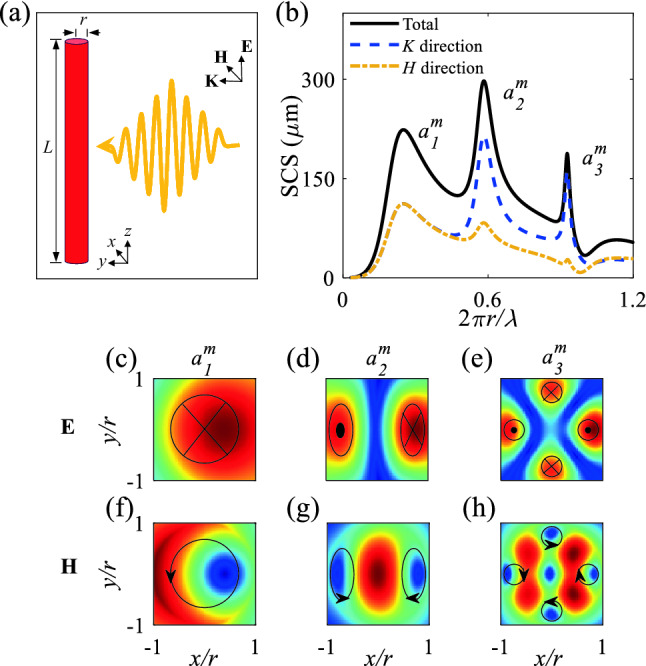
(**a**) Illumination of a single Ge rod with a refractive index *n* = 4, radius *r*, and length *L* ($L \gg r$) under a TM polarized plane wave (electric field along the long axis). (**b**) SCS of a long single Ge rod under TM polarized plane wave versus normalized frequency. (**c**–**e**) and (**f**–**h**) show E- and H-field distributions, respectively, of the rod for the Mie resonances of $a_{1}^m$, $a_{2}^m$, and $a_{3}^m$. Blue to red show minimum to maximum of the fields.

When a long Ge rod ($L \gg r$) is illuminated by a TM-polarized plane wave (Fig. 1a), the long *L* means that extraction of time and frequency (Fourier transform) responses of electric and magnetic fields requires 2D FDTD simulations. Simulation results of the scattering cross-section (SCS) show Mie resonance peaks at normalized frequency $\frac{2\pi r}{\lambda }$ = 0.254, 0.579, and 0.9225, respectively, which correspond to $a_{1}^{m}$, $a_{2}^{m}$, and $a_{3}^{m}$ (Fig. 1b). E-field distributions in the *xy* plane for $a_{1}^{m}$, $a_{2}^{m}$, and $a_{3}^{m}$ show one, two, and four electric dipoles, respectively, which originate from collective polarization of particle materials in response to the incident electric field (along the long axis of the rod) (Fig. 1c–e). The circulating magnetic fields inside the rod are created due to the oscillation of electric dipoles. The rod and the surrounding medium (air) are nonmagnetic, so the permeability $\mu _{r} = 1$; therefore, the magnetic current loops penetrate to the air (Fig. 1f–h).

Calculations of the total, *K*-direction, and *H*-direction SCSs of the Ge rod (Fig. 1b) show that $a_{1}^m$ represents the higher amplitude of the SCS in the *H* direction and that $a_{2}^m$, and $a_{2}^m$ reveal a stronger amplitude of SCS $a_{3}^m$ in that direction. The higher amplitude of SCS along *H* direction than the propagation direction (*K* direction) is responsible for a H-field coupling between rods that are in a periodic arrangement in that direction.

In a periodic arrangement of Ge rods in air with $r = 0.3a$ (where *a* is the lattice constant), excitation $a_{1}^m$, $a_{2}^m$, and $a_{3}^m$ modes induce the development of H-field couplings between rods located along the H-field direction, as a consequence of the induced circulating magnetic field inside each rod. These couplings suppress the incident wave to penetrate the periodic structure and result in bandgaps in the photonic band structure. In the periodic arrangement, the Mie resonances of $a_{1}^m$, $a_{2}^m$, and $a_{3}^m$ are responsible for creating Mie bandgaps of ${\mathrm{TM}}_{01}$, ${\mathrm{TM}}_{11}$, and ${\mathrm{TM}}_{21}$, respectively.

**Figure 2:**
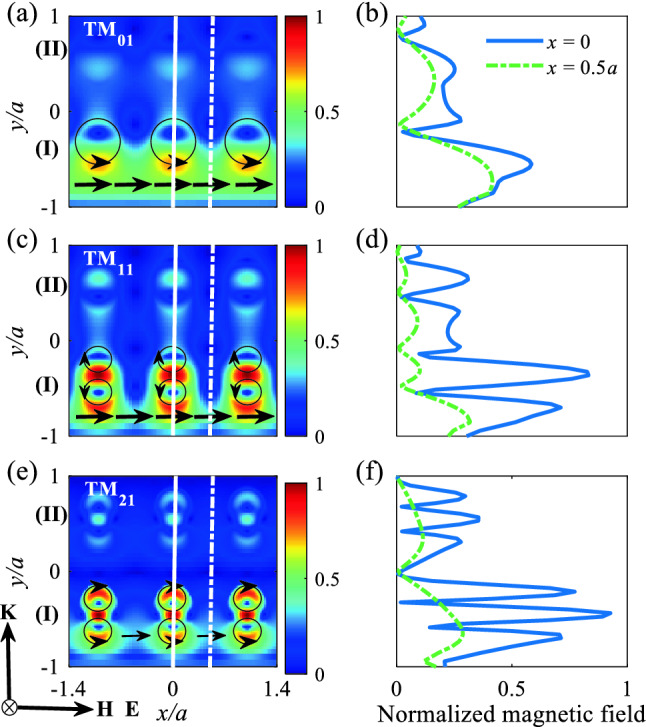
Periodic arrays of Ge rods in air under excitation of (**a**) ${\mathrm{TM}}_{01}$, (**c**) ${\mathrm{TM}}_{11}$ and (**e**) ${\mathrm{TM}}_{21}$ Mie bandgaps. (**b**), (**d**) and (**f**) Normalized magnetic fields for (**a**), (**c**), and (**e**), respectively, over *y* axis for two lines of $x = 0$ and $x = 0.5a$. Incident plane has been located at the bottom of the structure and propagates from $-y$ to *y* direction. Black arrows represent the direction of magnetic fields.

The intensity of the H-field couplings is proportional to the scattering along the H-field direction (Fig. 1b, *H* direction), consequently, the strengths of the H-field couplings between rods descend in order $a_{1}^m> a_{2}^m > a_{3}^m$. These couplings are depicted by H-field distributions as well as normalized H-field along $x = 0$ and $x = 0.5a$ lines (Fig. 2). In this figure, TM polarized plane waves which are located at the bottom of the structure propagate from $-y$ to *y* direction. H-fields of Mie resonances of $a_{1}^m$, $a_{2}^m$, and $a_{3}^m$ that are responsible for Mie bandgap modes of ${\mathrm{TM}}_{01}$, ${\mathrm{TM}}_{11}$, and ${\mathrm{TM}}_{21}$ have different distributions (Fig. 2a, c, e), and different normalized magnetic fields along $x = 0.5$ (green dotted-dashed line) and $x = 0$ (solid-blue line) (Fig. 2b, d, f). The incident plane waves that propagate from $-y$ to *y*, interact mostly with the first row (I) of the periodic Ge MMs rather than with the second row (II). These interactions can be shown quantitatively using normalized magnetic fields (Fig. 2b, d, f). The Mie scattering amplitude along the H-field direction is highest in $a_{1}^m$, so the H-field couplings between the first row of rods (I) (Fig. 2b, green broken line) descend in strength in the order $a_{1}^m> a_{2}^m > a_{3}^m$. The normalized magnetic fields in Fig. 2b, d, f at $x = 0$ (solid blue) for the first row of Fig. 2a, c, e, show one, two, and three peaks that are created due to the shape mode of $a_{1}^m$, $a_{2}^m$, and $a_{3}^m$, respectively. The created shape mode of magnetic fields inside each rod for ${\mathrm{TM}}_{01}$ and ${\mathrm{TM}}_{11}$ (Fig. 2a, c) are the same as $a_{1}^m$ and $a_{2}^m$ (Fig. 1f, g), respectively. Due to the H-field couplings between rods along the incident H-field, the shape mode inside each rod for ${\mathrm{TM}}_{21}$ (Fig. 2e) mode differs slightly from the shape mode of $a_{3}^m$ (Fig. 1h).

As described earlier the H-field couplings between rods are responsible for the creation of ${\mathrm{TM}}_{01}$, ${\mathrm{TM}}_{11}$, and ${\mathrm{TM}}_{21}$ bandgap modes; also, there are other ways that describe the creation of a photonic bandgap which are originated from solid-state physics. The creation of electronic bandgap in electronic crystals is explained by band or bond theory^43^. The band theory uses the Bloch theorem under perfect periodicity of the lattice structure which results in the creation of the Bragg bandgap. In contrast, bond theory explains the creation of the bandgap in periodic and amorphous electronic crystals by postulating the internal states of bonding and antibonding states in two adjacent atoms which result in the formation of the lower and upper bands of the bandgap. The bond theory can be used to explain the creation of photonic bandgap in disordered all-dielectric MMs. This theory postulates the coupling of optical states between two adjacent dielectric meta-atoms. The optical state in a dielectric meta-atom forms a quasi-bond state. The coupling of quasi-bond states between two adjacent dielectric meta-atoms forms an optical bandgap. In disordered dielectric MMs, the bond theory is the origin of the creation of Mie bandgaps that robust disordering^44^.

### Position disordering

Mie bandgaps normally appear as dips in transmission diagrams of the periodic arrangement of high-*n* dielectric rods, because of a transition from PCs to all-dielectric MMs. This part demonstrates that the Ge MMs show ${\mathrm{TM}}_{01}$ and ${\mathrm{TM}}_{11}$ bandgaps that tolerate significant disordering of the rod position. The position of a rod in a periodic structure is defined as ($x^{i}, y^{i}$), where $x^{i}=x_{0}^i+\sigma _{p}U_{x}$ and $y^{i}=y_{0}^i+\sigma _{p} U_{y}$. ($x_{0}^i,y_{0}^i$), is the origin position of the rod in the periodic structure, $\sigma _{p}$ is the strength of the position disordering, and $U_{i}$(*i* : *x*, *y*), are random variables over interval [− 1, 1] along *x* and *y* directions with a uniform random distribution, respectively. The position-disordering parameter is defined as $\eta _{p}=\frac{\sigma _{p}}{a}$, where *a* is the lattice constant^29^.

**Figure 3:**
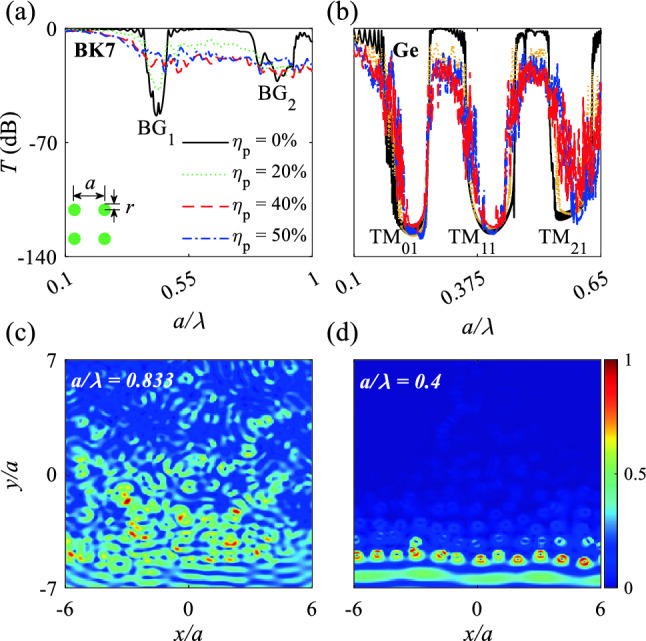
(**a**) and (**b**) logarithmic transmission spectra of BK7 PCs and Ge MMs in a cubic pattern under TM polarized plane waves (H field along *x* direction) for position disordering $\eta _{p}$ = 0, 20, 40, and 50%. (**c**) and (**d**) H-field distributions of BK7 PCs and Ge MMs at the center of the Bragg bandgap ${\mathrm{BG}}_{2}$ ($\frac{a}{\lambda }=0.833$) and Mie bandgap ${\mathrm{TM}}_{11}$ ($\frac{a}{\lambda } = 0.41$) under $\eta _{p}$ = 40%. The plane waves propagate from $-y$ to *y* direction.

Transmission spectra under illumination of TM-polarized plane waves (magnetic field is along *x* direction) were obtained for borosilicate crown glass (BK7) PCs in air (Fig. 3a) and Ge MMs in air (Fig. 3b). BK7 PCs were composed of cylindrical BK7 rods with $n = 1.5$ and Ge MMs were composed of Ge rods with $n = 4$; both types were set in a periodic-cubic pattern under rod-position disordering of $\eta _p$ = 0, 20, 40, or 50%. Incident plane waves are located at the bottom of the structures, propagate from $-y$ to *y* direction, and are monitored at the top of the structures (Figs. 3c, d and  6c, d).

The BK7 PCs showed two week Bragg bandgaps of ${\mathrm{BG}}_{1}$ and ${\mathrm{BG}}_{2}$ over $0.1<\frac{a}{\lambda }<1$ that do not tolerate position disordering (Fig. 3a). At $\eta _{p}$ = 0 the Bragg bandgaps of ${\mathrm{BG}}_{1}$ and ${\mathrm{BG}}_{2}$ appear at $\frac{a}{\lambda }=$ 0.44 and 0.87, respectively and disappear with increase in $\eta _{p}$. The Bragg bandgap obeys $f\propto (acos^{-1}(\theta ))$, where $\theta$ is the propagation angle. Increase in position disordering changes both *a* and $\theta$, so the Bragg bandgaps diminish and disappear. Also, position disordering breaks the symmetric of the periodic structure which results in degradation of the Bragg bandgaps. H-field distributions of ${\mathrm{BG}}_{2}$ show penetration of TM polarized plane wave at $\frac{a}{\lambda }=0.833$ which results in degradation of ${\mathrm{BG}}_{2}$ (Fig. 3c).

The Ge structure showed three Mie bandgaps ${\mathrm{TM}}_{01}$, ${\mathrm{TM}}_{11}$, and ${\mathrm{TM}}_{21}$, of which ${\mathrm{TM}}_{01}$ and ${\mathrm{TM}}_{11}$ reveal strong position disordering that can tolerate $\eta _p = 50\%$ with average $T = -135$ dB. The strong tolerance of the Mie bandgaps of ${\mathrm{TM}}_{01}$ and ${\mathrm{TM}}_{11}$ under position disordering occurs for two reasons. The first is that the Ge rods have high SCSs $a_{1}^m$ and $a_{2}^m$ along with incident H-field direction, so adjacent rods develop high H-field coupling. The second reason is that ${\mathrm{TM}}_{01}$ and ${\mathrm{TM}}_{11}$ are pure Mie bandgaps and show high tolerance to position disordering, whereas ${\mathrm{TM}}_{21}$ is a mixture of Bragg and Mie bandgaps which degrades with increase in position disordering. The H-field distribution of BK7 PCs at the Bragg bandgap ${\mathrm{BG}}_{2}$ ($\frac{a}{\lambda }=0.833$) show diffusion of electromagnetic waves all over the structure, whereas the H-field distribution of Ge MMs for the ${\mathrm{TM}}_{11}$ Mie bandgap ($\frac{a}{\lambda }=0.4$) mode show suppression of incident waves by each rod of the first row. An increase in position disordering, increases the distance between some rods along *x* and *y* directions which results in declining the coupling between the rods as well as weakens coupling between optical quasi-bond states which in turn narrows bandwidths of ${\mathrm{TM}}_{01}$ and ${\mathrm{TM}}_{11}$.

#### Position-disorder-tolerant Ge narrow straight, L-shaped and crossing waveguides

Ge MMs tolerate intense disorder of $\eta _p=50\%$ for ${\mathrm{TM}}_{01}$ and ${\mathrm{TM}}_{11}$ Mie bandgaps under TM polarized plane wave (Fig. 3b). This high tolerance of position disordering is a result of Mie scattering by individual rods in the first row of the structure (Figs. 2, 3d); therefore, the first row (I) has a significant effect on suppression of incident TM polarized light, and also, localization of H-field inside each rod of the first row.

**Figure 4:**
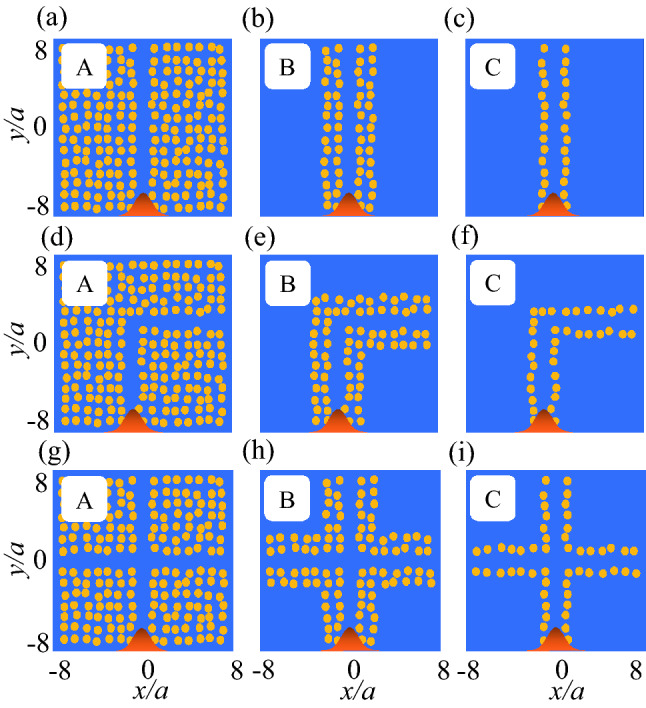
All-dielectric straight, L-shaped, and crossing waveguides surrounded by (**a**), (**d**), and (**g**) 14, (**b**), (**e**), and (**h**) four, and (**c**), (**f**), and (**i**) two rows of dielectric rods in air that are called A, B, and C structures. The dielectric rods are either BK7 or Ge. The waveguides are under $\eta _p = 20\%$. Yellow: dielectric rod; blue: air. An incident Gaussian sources located at the bottom of the structures and propagate from $-y$ to *y* direction.

To exploit this effect, we suggest ultra-narrow straight, L-shaped, and crossing waveguides that contain 14, four or two rows of rods, and that tolerate a specific degree of position disordering. For this purpose, straight, L-shaped, and plus shaped defects were surrounded by 14 (Fig. 4a, d, g; structure ‘A’), four (Fig. 4b, e, h; structure ‘B’), or two (Fig. 4c, f, i; structure ‘C’) rows of dielectric rods (either BK7 or Ge) in air. Creating straight, L-shaped, or plus-shaped defect inside the Ge MMs localizes guided modes within the Mie bandgaps of ${\mathrm{TM}}_{01}$, ${\mathrm{TM}}_{11}$, and ${\mathrm{TM}}_{21}$ (Fig. S1) that are called donor modes. For the BK7 PCs, the line defect localizes guided modes inside the Bragg bandgaps of ${\mathrm{BG}}_{1}$ and ${\mathrm{BG}}_{2}$ (Fig. S1). The guided mode inside the ${\mathrm{TM}}_{01}$ bandgap propagates inside the rods by H-field coupling, but for ${\mathrm{TM}}_{11}$ this guided mode acts as a total-internal-reflection effect and propagates through the waveguides; therefore, ${\mathrm{TM}}_{11}$ is exploited for Ge waveguides. Incident Gaussian waves located at the bottom of the structures in an orange bell-shaped, propagate from -*y* to *y* direction and are monitored at the top (straight and crossing waveguides) and right (L-shaped waveguides) parts of the structures (Fig. 4).

**Figure 5:**
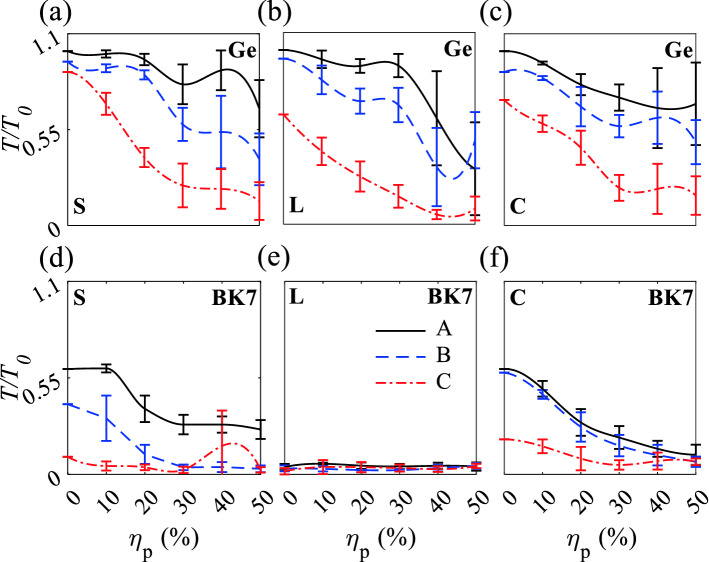
Normalized transmission versus $\eta _{p}$ for A, B, and C straight, L-shaped, and crossing waveguides. (**a**)–(**c**) and (**d**)–(**f**) Ge and BK7 waveguides. Gaussian sources are lunched at the bottom of the waveguides, propagate from $-y$ to *y* direction. S, L, and C stand for straight, L-shaped and crossing waveguides, respectively.

Transmission spectra for the Ge and BK7 waveguides are monitored for the localized mode inside the ${\mathrm{TM}}_{11}$ and ${\mathrm{BG}}_{2}$, respectively. In the Ge straight, L-shaped and crossing waveguides, the transmissions are higher and show robustness at all $\eta _p$ than for BK7 PCs (Fig. 5). Ge-based A-type straight, L-shaped and crossing waveguides have high average normalized transmission amplitude ($\frac{T}{T_0}$) $> 96, 90,$ and $80\%$, respectively at $\eta _p \le 20\%$; also, show the small variation of the average $\frac{T}{T_0}$. Ge-based B-type straight, L-shaped and crossing waveguides have average $\frac{T}{T_0} > 86, 70,$ and $70\%$ at $\eta _p \le 20\%$ and C-type straight, L-shaped and crossing waveguides show average transmission $> 70, 40,$ and $46\%$ at $\eta _p \le 10\%$ (Fig. 5a–c). H-field distributions of B-type waveguide under $\eta _p = 20\%$ show high coupling of incident light waves to the outputs as well as represent robustness of $\frac{T}{T_0}$ to the sharp bend in a L-shaped waveguide and a horizontal defect in a crossing waveguide (Fig. S2a–c). Decreasing the number of rows of Ge rods from A-type waveguides surrounded by 14 rows, to B-type waveguides with four rows decreased the average transmission slightly (Fig. 5a–c). The decreases in average $\frac{T}{T_0}$ at $\eta _p > 20\%$ for B waveguides and at $\eta _p > 10\%$ for C waveguides, occurs because some rods are separated by large distances, which result in H-field coupling degradation, fleeing electromagnetic waves, and a from guided mode to radiative mode; also the increase in distances decreases the coupling between quasi-bond states between rods and results in escaping of electromagnetic waves.

In the BK7 PCs straight, L-shaped and crossing waveguides, the average $\frac{T}{T_0}$ is much less than the Ge MMs (Fig. 5d–f), owning to the lower refractive index. BK7-based A-type straight and crossing waveguides have average $\frac{T}{T_0} < 0.6$ at $\eta _p \le 10\%$ and the average $\frac{T}{T_0}$ decreased with increase in position disordering. Reducing the number of BK7 rods in straight and crossing waveguides from 14 (type A) to four (type B) and two (type C) decreases the average $\frac{T}{T_0}$, owning to the dependence of defect mode inside the Bragg bandgap the is strongly dependent to the periodicity and the number of unit cell of the structure. Because of sharp bend in BK7 based L-shaped waveguides, the average $\frac{T}{T_0}$ is about zero for A, B, and C-type waveguides. The guided mode in L-shaped waveguides turn into the radiation mode at the sharp bend due to the low refractive index of BK7 which is represented in H-field distribution of B-type waveguides at $\eta _p = 20\%$ (Fig. S2d–f). Also, by increasing the position disordering, the average normalized transmission amplitudes decrease sharply due to the breaking symmetry in the structure. As obvious, the average $\frac{T}{T_0}$ of L-shaped BK7 waveguides (Fig. 5e) is less than the L-shaped Ge-based waveguides (Fig. 5b) that implies the robustness of ${\mathrm{TM}}_{11}$ in Ge-based to sharp bends.

### Radius disordering

The effect of radius disordering can also be exploited in the transition from BK7 PCs to Ge MMs. Under radius disordering, the radius is $R^i=R_i^0+\sigma _r U$, where $R_i^0$ is the origin radius, $\sigma _r$ is the strength of the radius disordering, and *U* is a random variable over the interval [− 1, 1]. The radius-disordering parameter is defined as $\eta _r=\sigma _r/R_i^0$.

**Figure 6:**
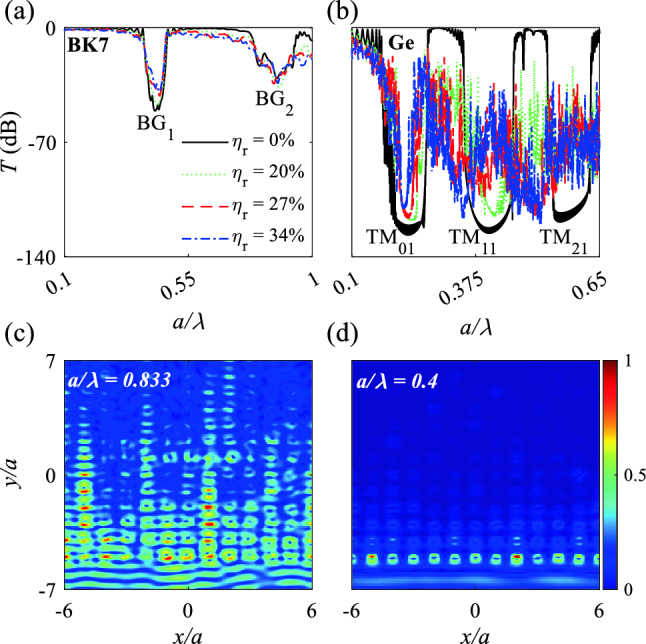
(**a**) and (**b**) logarithmic transmission spectra of BK7 PCs and Ge MMs in a cubic pattern under TM polarized plane waves (H field along *x* direction) for radius disordering $\eta _{r}$ = 0, 20, 27, and 34%. (**c**) and (**d**) H-field distributions of BK7 PCs and Ge MMs at the center of the Bragg bandgap ${\mathrm{BG}}_{2}$ ($\frac{a}{\lambda }=0.833$) and Mie bandgap ${\mathrm{TM}}_{11}$ ($\frac{a}{\lambda } = 0.41$) under $\eta _{r}$ = 27%. The plane waves propagate from $-y$ to *y*.

BK7 PCs show weak Bragg bandgaps of ${\mathrm{BG}}_{1}$ and ${\mathrm{BG}}_{2}$ with $T = -50$ and $-25$ dB, respectively, that are tolerant of $\eta _r = 20\%$, because the periodic lattice is retained under radius disordering (Fig. 6a). For Ge MMs under radius disordering, the radii of the rods change, so their localized mode changes; these changes degraded the ${\mathrm{TM}}_{21}$ Mie bandgap but ${\mathrm{TM}}_{11}$ and ${\mathrm{TM}}_{01}$ tolerated $\eta _r=20$ and 34%, respectively (Fig. 6b), because the ${\mathrm{TM}}_{01}$ and ${\mathrm{TM}}_{11}$ mode shapes inside each rod do not change at less than a certain $R^{i}$.

As $\eta _r$ increased, the width of the ${\mathrm{TM}}_{01}$ and ${\mathrm{TM}}_{11}$ Mie bandgaps decreased, as a result of light penetration through rods with small radii, and the penetrated light waves in turn experience interferences that result in high oscillation in transmission spectrum around ${\mathrm{TM}}_{01}$, ${\mathrm{TM}}_{11}$, and ${\mathrm{TM}}_{21}$ Mie bandgaps (Fig. 6b). H-field distribution for BK7 PCs and Ge MMs at the Bragg bandgap ${\mathrm{BG}}_{2}$ ($\frac{a}{\lambda }=0.833$) (Fig. 6c) and ${\mathrm{TM}}_{11}$ Mie bandgap ($\frac{a}{\lambda }=0.4$)(Fig. 6d) show the leaking of H-field distribution for Bragg bandgap and suppression of the incident field for the ${\mathrm{TM}}_{11}$ bandgap by the individual Ge rods of the first row.

#### Radius-disorder-tolerant Ge narrow straight, L-shaped and crossing waveguides

**Figure 7:**
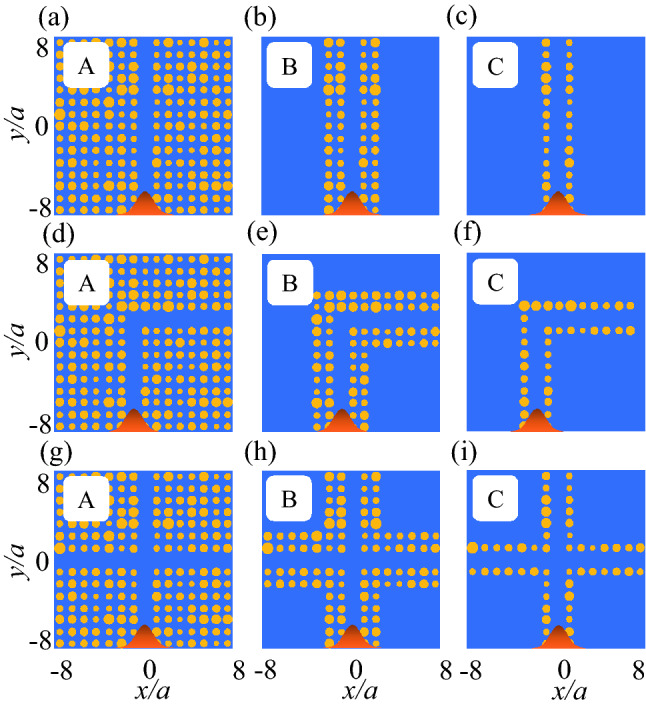
All-dielectric straight, L-shaped, and crossing waveguides surrounded by (**a**), (**d**), and (**g**) 14, (**b**), (**e**), and (**h**) four, and (**c**), (**f**), and (**i**) two rows of dielectric rods in air that are called A, B, and C structures. The waveguides are under $\eta _r = 27\%$. The dielectric rods are either BK7 or Ge. Yellow: dielectric rod; blue: air. An incident Gaussian sources located at the bottom of the structures and propagate from $-y$ to *y* direction.

As a result of the high-*n* elements of Ge MMs, the ${\mathrm{TM}}_{01}$ and ${\mathrm{TM}}_{11}$ Mie bandgaps tolerate radius disordering of $\eta _r$ = 34 and 20%, respectively (Fig. 6b). We exploited this tolerance to design ultra-narrow Ge straight, L-shaped and crossing waveguides that can endure radius disordering to a certain level. In this section the Ge MMs waveguides that use 14 (Type A waveguide), four (Type B), or two (Type C) rows of Ge rods are designed and simulated. The three types of Ge waveguides were analyzed under radius disordering (Fig. 7).

**Figure 8:**
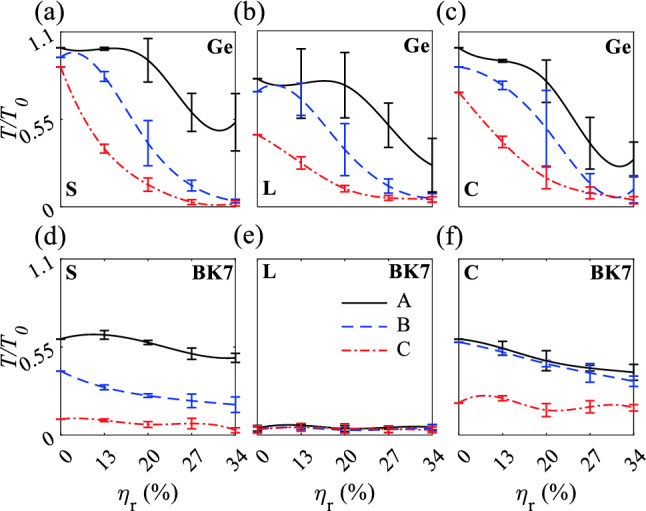
Normalized transmission versus $\eta _{r}$ for A, B, and C straight, L-shaped, and crossing waveguides. (**a**)–(**c**) and (**d**)–(**f**) Ge and BK7 waveguides. Gaussian sources are lunched at the bottom of the waveguides, propagate from $-y$ to *y* direction. S, L, and C stand for straight, L-shaped and crossing waveguides, rspectively.

Creating straight, L-shaped or plus-shaped defect localizes guided modes inside the Mie and Bragg bandgaps (Fig. S1). The average *T* for the Ge waveguides increased as the number of rows of rods increased, but decreased as $\eta _r$ increased (Fig. 8). Waveguides A tolerated strong radius disorder of $\eta _r \le 20\%$ with average $\frac{T}{T_0} > 0.93, 0.76,$ and 0.8, but under $\eta _r > 20\%$, $\frac{T}{T_0}$ decreased gradually. Due to the straight path for light waves for A-type straight and crossing waveguides, the $\frac{T}{T_0}$ has small variation at $\eta _r<20\%$. Waveguides B tolerated $\eta _r \le 13\%$ with average $\frac{T}{T_0} > 0.82, 0.67,$ and 0.76 for straight, L-shaped, and crossing waveguides, respectively. H-field distributions of B-type waveguides at $\eta _r = 13\%$ represent high confinement of light waves in the waveguides and robustness of the confinement to the sharp bend and horizontal defect in L-shaped and crossing waveguides (Fig. S3a–c). Also, $\frac{T}{T_0}$ has larger variation at $\eta _r<20\%$, owning to the sharp bend in L-shaped waveguide A and B. Waveguide C show lower toleration to radius disordering. There are three reasons governing decreasing average $\frac{T}{T_0}$ for Ge-based waveguides under radius disordering. First, increasing radius disordering decreases some radii of rods that increases the distances between them and decreases H-field coupling between them and change the guided mode to radiation mode which result in decreasing the average $\frac{T}{T_0}$. The second reason is diminishing quasi-bond states couplings between rods which have larger distances due to the radius disordering; the weaker coupling results in escaping electromagnetic waves from the waveguides. The last reason originated from non-localization of ${\mathrm{TM}}_{11}$ mode inside the rods due to the different radii.

BK7 straight, L-shaped, and crossing waveguides have much lower transmission than Ge waveguides (Fig. 8d–f). The BK7 straight and crossing waveguides show higher transmission than L-shaped wavegudies. The low transmission of L-shaped wavegudies are originated from a sharp bend in the structure that can not confine light waves due to the low refractive index of BK7 as shown in Fig. S3d–e.

### Comparison between TM and TE modes in Te, Ge, and Si MMs

This part describes the existence of ${\mathrm{TM}}_{01}$, ${\mathrm{TM}}_{11}$, ${\mathrm{TM}}_{21}$, ${\mathrm{TM}}_{02}$, ${\mathrm{TM}}_{12}$ and ${\mathrm{TM}}_{01}$ Mie bandgaps in periodic and disordered structures contain dielectric rods (either Te, Ge, or Si) in air. Te, Ge, and Si are non-dispersive materials over 4 $\upmu$m $< \lambda<$ 10 $\upmu$m, 2 $\upmu$m $< \lambda<$ 10 $\upmu$m, and 2 $\upmu$m $< \lambda<$ 10 $\upmu$m, respectively (Fig. S5). In contrast to the ${\mathrm{TM}}_{01}$ Mie bandgap mode that appear in all-dielectric MMs contain high-refractive index elements^29,30^, ${\mathrm{TM}}_{01}$, ${\mathrm{TM}}_{11}$, ${\mathrm{TM}}_{21}$ Mie bandgaps appear in all-dielectric MMs contain lower refractive index elements such as Si as numerically simulated in the following.

**Figure 9:**
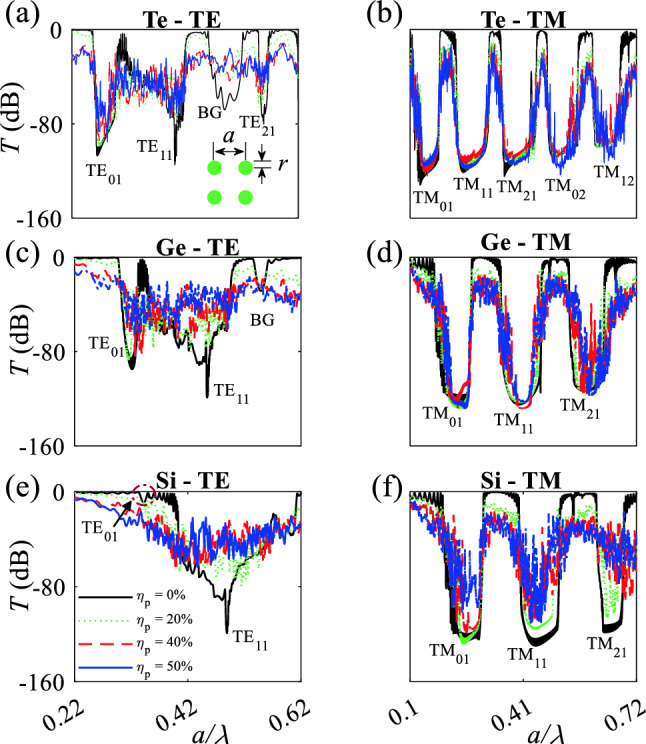
Logarithmic transmission spectra of Te MMs contain $15\times 15$ Te rods in air under illumination of TE (**a**) and TM (**b**) plane waves, respectively. The spectra of $15\times 15$ Ge rods in air under illumination of TE (**c**) and TM (**d**) plane waves, respectively. (**e**) and (**f**) show the spectra of $15\times 15$ Si rods in air under illumination of TE and TM polarized plane waves, respectively. Solid black, dotted green, dashed red, and dot-dashed blue curves represent the position disordering of $\eta _p = 0, 20, 40$, and $50\%$, respectively.

Te is an anisotropic material with refractive indices under TE and TM polarized waves of $n_{0} = 4.8$ and $n_{e} = 6.2$ over a wavelength range of 4 $\upmu$m $< \lambda<$ 10 $\upmu$m (Fig. S5). The Te MMs’ structure consist of $15\times 15$ Te rods in air under TE polarized light reveals a ${\mathrm{TM}}_{01}$ Mie mode which just tolerates a rod-position disordering of $\eta _p= 20\%$ (Fig. 9a). The structure under the illumination of TM polarized waves shows five bandgaps of ${\mathrm{TM}}_{01}$, ${\mathrm{TM}}_{11}$, ${\mathrm{TM}}_{21}$, ${\mathrm{TM}}_{02}$, ${\mathrm{TM}}_{12}$ in which ${\mathrm{TM}}_{01}$, ${\mathrm{TM}}_{11}$, ${\mathrm{TM}}_{21}$, and ${\mathrm{TM}}_{02}$ reveal wide bandwidths and high robustness to the rod-position disordering of $\eta _p = 50\%$ (Fig. 9b). There are a few reasons behind the high robustness of ${\mathrm{TM}}_{01}$, ${\mathrm{TM}}_{11}$, ${\mathrm{TM}}_{21}$, and ${\mathrm{TM}}_{02}$ to position disordering. First, the SCSs of each Te rod along the H-field are high enough that guarantee the coupling of the H-field between adjacent rods results in position-disorder toleration. Also, the coupling is originated from quasi-bond states between rods. Second, the bands are pure Mie type and reveal robustness to position disordering, whereas ${\mathrm{TM}}_{21}$ is a mixture of Mie and Bragg bandgaps which weakens the tolerance to position disordering. The structure under rod-radius disordering and under illumination of either TE and TM modes show the strong and broadband TM Mie bandgaps and a narrow-weak ${\mathrm{TM}}_{01}$ Mie bandgap mode (Fig. S4a, b).

All-dielectric MMs consist of an array of $15\times 15$ Ge rods with $n = 4$ in air under illumination of a TE polarized plane wave show a weak and narrow ${\mathrm{TM}}_{01}$ at $0.3<\frac{a}{\lambda }<0.33$ which robust a low level of position disordering ($\eta _p = 20\%$); in addition an ultra-narrow ${\mathrm{TM}}_{11}$ Mie bandgap and a very weak Bragg bandgap (BG) that do not tolerate position disordering appear in the transmission spectra (Fig. 9c). In contrast, the structure under illumination of a TM plane wave shows three intense and wide Mie bandgaps of ${\mathrm{TM}}_{01}$ ($0.17<\frac{a}{\lambda }<0.266$), ${\mathrm{TM}}_{11}$ ($0.351<\frac{a}{\lambda }<0.461$), and ${\mathrm{TM}}_{21}$ ($0.53<\frac{a}{\lambda }<0.63$). ${\mathrm{TM}}_{01}$ and ${\mathrm{TM}}_{11}$ tolerate a position disordering $\eta _p = 50\%$ and ${\mathrm{TM}}_{21}$ tolerates a position disordering $\eta _p = 20\%$ (Fig. 9d). As mentioned earlier, the strong robustness of ${\mathrm{TM}}_{01}$ and ${\mathrm{TM}}_{11}$ is originated from the fact that they are pure Mie bandgaps while ${\mathrm{TM}}_{21}$ is a combination of Mie and bragg bandgaps that does not tolerate position disordering. The structure under radius disordering and illumination of either TE or TM modes show the strong and broadband TM Mie bandgaps and a narrow-weak ${\mathrm{TM}}_{01}$ Mie bandgap mode (Fig. S4c, d).

The ${\mathrm{TM}}_{01}$ Mie bandgap mode almost disappears in a MMs structure contains Si rods with $n = 3.48$ in air under illumination of TE polarized plane wave (Fig. 9e). In contrast, the Si MM structure under a TM polarized incident plane wave shows three strong and broadband of ${\mathrm{TM}}_{01}$, ${\mathrm{TM}}_{11}$, and ${\mathrm{TM}}_{21}$; furthermore, ${\mathrm{TM}}_{01}$ and ${\mathrm{TM}}_{11}$ reveal tolerant to position disordering $\eta _p = 40$ and 20$\%$, respectively (Fig. 9f). ${\mathrm{TM}}_{01}$ approximately disappears and show no robustness to radius disordering in Si MMs structure while three intense and broad bandgaps appear in which ${\mathrm{TM}}_{01}$ and ${\mathrm{TM}}_{11}$ show robustness to radius disordering $\eta _r = 34$ and $20\%$, respectively (Fig. S4e, f). Based on the results, ${\mathrm{TM}}_{01}$ only appears in Ge MMs and disappears in Si MMs, that shows highly dependent of the ${\mathrm{TM}}_{01}$ Mie mode to the refractive index; the dependent is due to the highly-dependence of TE Mie scattering to the refractive index. Intense and broadband ${\mathrm{TM}}_{01}$, ${\mathrm{TM}}_{11}$, and ${\mathrm{TM}}_{21}$ appear in Ge and Si MMs, which imply a very low dependence of Mie scattering modes of $a_{1}^{m}$, $a_{2}^{m}$, and $a_{3}^{m}$ to the refractive index.

### Robustness of ${\mathrm{TM}}_{01}$, ${\mathrm{TM}}_{11}$, and ${\mathrm{TM}}_{21}$ to rod-position and rod-radius disordering

**Figure 10:**
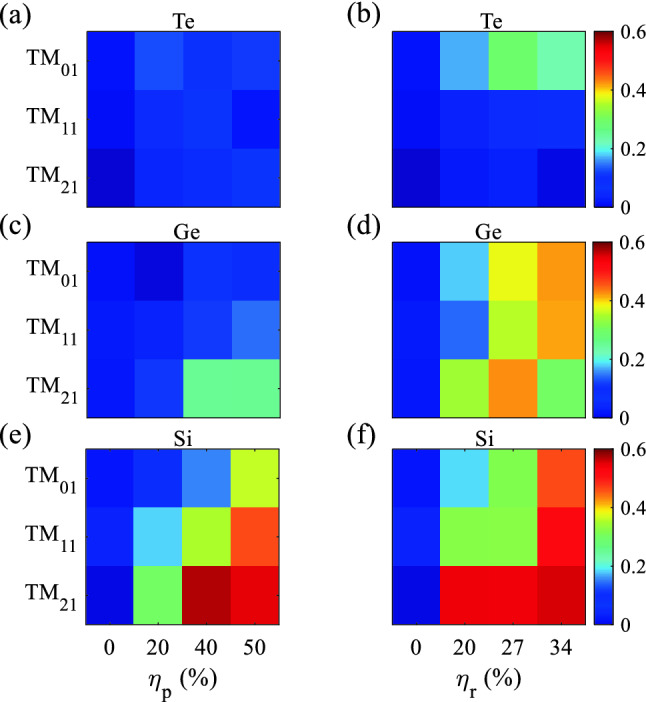
$\gamma$ parameter for ${\mathrm{TM}}_{01}$, ${\mathrm{TM}}_{11}$, and ${\mathrm{TM}}_{21}$ Mie bandgap modes for the structures contain Te (**a**), Ge (**c**), and Si (**e**) rods in air under rod-position disordering of $\eta _p = 0, 20, 40$, and 50%. $\gamma$ parameter for ${\mathrm{TM}}_{01}$, ${\mathrm{TM}}_{11}$, and ${\mathrm{TM}}_{21}$ Mie bandgap modes for the structures contain Te (**b**), Ge (**d**), and Si (**f**) rods in air under rod-radius disordering of $\eta _r = 0, 20, 27$, and 34%.

To illustrate the robustness of Te, Ge, and Si MMs to rod-position and rod-radius disordering, the parameter $\gamma =\frac{(\overline{R_0}-\overline{R})}{\overline{R_0}}$ is defined. Where $\overline{R} = \int _{\lambda _1}^{\lambda _2}\frac{Rd\lambda }{(\lambda _2 - \lambda _1)}$, $\overline{R_0}$, and $\lambda _i (i:1,2)$ are average reflection over the bandgap, the reflection of the bandgap with zero disordering, and bandgap wavelengths at 10% of the transmission, respectively. The $\gamma$ parameter for three Mie bandgaps of ${\mathrm{TM}}_{01}$, ${\mathrm{TM}}_{11}$, and ${\mathrm{TM}}_{21}$ for Te, Ge, and Si MMs under rod-radius and rod-position disordering shows the transition of Mie to Mie + Bragg and Bragg bandgaps (Fig. 10). $\gamma <0.25$, $0.25<\gamma <0.3$, and $\gamma >0.3$ are categorized as Mie, Mie + Bragg, and Bragg bandgaps, respectively. ${\mathrm{TM}}_{01}$, ${\mathrm{TM}}_{11}$, and ${\mathrm{TM}}_{21}$ Mie bandgaps of Te MMs reveal the highest robustness to a rod-position disordering of $\eta _p=50\%$ (Fig. 10a, b). Although, the Mie bandgaps show rod-radius robustness of $\eta _r=34\%$, the bandgaps’ edges disappear except for ${\mathrm{TM}}_{01}$ (Fig. 10b). ${\mathrm{TM}}_{01}$ and ${\mathrm{TM}}_{11}$ for Ge MMs show rod-position robustness of $\eta _p=50\%$ and ${\mathrm{TM}}_{01}$ reveals rod-radius robustness of $\eta _r=34\%$ with the narrower bandwidth (Fig. 10c, d). As obvious, the ${\mathrm{TM}}_{01}$ in Si-based MMs shows rod-position robustness of $\eta _p=40\%$, and rod-radius disordering of $\eta _r=34\%$ with an inevitable cost of bandwidth narrowing (Fig. 10e, f). In conclusion, increasing the refractive index from Si to Ge and then Te increased the robustness of the MMs to rod-position and rod-radius disordering for TM Mie bandgap modes.

It is worth mentioning that the MMs contain dielectric rods of either Te, Ge, or Si illustrate three-wide bandgaps of ${\mathrm{TM}}_{01}$, ${\mathrm{TM}}_{11}$, and ${\mathrm{TM}}_{21}$, while the TE counterpart shows just one ${\mathrm{TM}}_{01}$ bandgap. ${\mathrm{TM}}_{01}$ and ${\mathrm{TM}}_{11}$ for Te and Ge MMs under rod-position disordering show pure Mie bandgaps; for Te and Ge MMs, ${\mathrm{TM}}_{21}$ is Mie and Mie + Bragg bandgaps, respectively (Fig. 10a, c). Si MMs under rod-position disordering show Mie + Bragg bandgap of ${\mathrm{TM}}_{01}$ and Bragg bandgap of ${\mathrm{TM}}_{11}$ and ${\mathrm{TM}}_{21}$ (Fig. 10e). ${\mathrm{TM}}_{01}$ under rod-radius disordering show the transition from Mie to Mie + Bragg and to Bragg for Te, Ge, and Si MMs, respectively (Fig. 10b, d, f). ${\mathrm{TM}}_{11}$ and ${\mathrm{TM}}_{21}$ under rod-radius disordering depict Mie bandgaps with an disappear bandgaps’ edges for Te MMs (Fig. 10b). ${\mathrm{TM}}_{11}$ and ${\mathrm{TM}}_{21}$ under rod-radius disordering reveal Bragg bandgaps (Fig. 10d, f).

## Conclusion

The paper has revealed the tolerance of Ge MMs to disordering of the position and radius of the rods that constitute robustness of the MMs to fabrication imperfections. This tolerance is a result of directive scattering of light waves by Te, Ge, and Si rods in air; this scattering induces H-field couplings between adjacent rods. ${\mathrm{TM}}_{01}$, ${\mathrm{TM}}_{11}$, and ${\mathrm{TM}}_{21}$ appear in Te MMs that tolerate position disordering $\eta _p = 50\%$ and radius disordering $\eta _r$ = 34, 27%, and 20%, respectively. The ${\mathrm{TM}}_{01}$ and ${\mathrm{TM}}_{11}$ Mie bandgap modes of Ge structures tolerate position disordering $\eta _p = 50\%$ and radius disordering $\eta _r$ = 34 and 20%, respectively. Rod-position and rod-radius disordering were exploited to design ultra-narrow straight, L-shaped, and crossing waveguides composed of 14, four, and two rows of Ge rods in air. These waveguides show average $\frac{T}{T_0}> 0.96, 0.9,$ and 0.8 for type A and $\frac{T}{T_0}> 0.86, 0.7,$ and 0.7 for type B at $\eta _p \le 20\%$. Also, These waveguides show average $\frac{T}{T_0}> 0.93, 0.76,$ and $0.8\%$ for type A at at $\eta _p \le 20\%$ and $\frac{T}{T_0}> 0.96, 0.9,$ and $0.8\%$ for type B at $\eta _r \le 13\%$. In addition, intense TM modes emerge in lower *n* (Si) MMs that tolerate a certain level of disordering; in contrast, ${\mathrm{TM}}_{01}$ fades in Si MMs. The tolerances to position and radius disordering of Te, Ge, and Si MMs provide a resource for mass production of photonic components that are ultra-narrow and robust to fabrication imperfections, for use in high-density OICs.

## Supplementary Information

Supplementary Information.

## Data Availability

All data generated or analysed during this study are included in this published article [and its supplementary information files].
