# Supplementary material for: Super strong wide TM Mie bandgaps tolerating disorders
Source: Sci Rep. 2022 May 12;12:7884. doi: 10.1038/s41598-022-11610-0 (PMC9098900; doi:10.1038/s41598-022-11610-0)
Supplement: Supplementary file 1 — Supplementary Information. [file 41598_2022_11610_MOESM1_ESM.pdf]

Super strong wide TM Mie bandgaps tolerating disorders

Kiyanoush Goudarzi*[1] and Moonjoo Lee*[1]

[1]Department of Electrical Engineering, Pohang University of Science and Technology (POSTECH), 37673 Pohang, Korea
*goudarzi@postech.ac.kr *and* moonjoo.lee@postech.ac.kr

ABSTRACT

The supplementary material proposes transmission spectra of localized modes inside the Ge-based waveguides under no rod-position and rod-radius disordering and H-field distribution for the Ge-based B-type waveguides under $\eta_p = 20\%$ and $\eta_r = 13\%$. Also, TE and TM Mie bandgaps and material dispersion spectra for Te, Ge and Si metamaterials under the radius disordering are studied.

Localized modes

Creating straight, L-shaped, and plus shaped defects by removing dielectric rods (BK7 or Ge) localize donor modes inside the Mie and Bragg bandgaps. The defects decrease the effective refractive index of the medium; as a result, donor modes appear in the bandgaps. The donor modes come from the upper edge of the bandgaps. Transmission (T) spectra of BK7 PCs under TM polarized plane waves at $\eta_r = \eta_p = 0$ shows two bragg bandgaps of BG_1 and BG_2 (Fig. S1(a)), while for the Ge MMs, T shows three Mie bandgap dips of TM_{01}, TM_{11}, and TM_{21} (Fig. S1(b)). The difference between the number of modes is originated from different refractive index of Ge and BK7. For the BK7 PCs of A-type straight and crossing waveguides, $T = -4$ dB at $0.79 < \frac{a}{\lambda} < 0.92$ (BG_2), while for A-type L-shaped waveguide $T = -27$ dB. The low T of the L-shaped waveguide is originated form 90° bend in the structure.

Transmission spectra under the same condition for Ge MMs for A-type of straight and crossing waveguides show $T \approx 0$ dB, and for the L-shaped waveguide $T \approx -10$ dB at $0.25 < \frac{a}{\lambda} < 0.27$ (TM_{01}). For the localized mode inside the TM_{11}, $T \approx 0$ dB at $0.37 < \frac{a}{\lambda} < 0.46$; also, for TM_{21}, T shows a variation at $0.53 < \frac{a}{\lambda} < 0.63$. The oscillations of modes in the Mie bandgaps are owning to the constructive and destructive interference of the guided waves to the structure's rods. T has the highest value over TM_{11} that is suitable to design the waveguides.

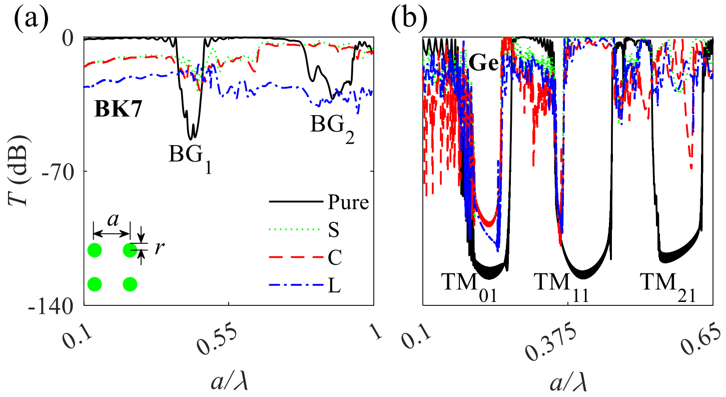

Fig. S1. (a) and (b) show logarithmic transmission spectra of BK7 PCs and Ge MMs in a cubic pattern under TM polarized plane waves (H field along x direction) for pure lattice and A-type structure of straight (S), L-shaped (L), and crossing (C) waveguides at $\eta_p = \eta_r = 0\%$. For numerical calculation $r = 0.3a$.

H-field distributions

H-field distributions for the B-type waveguides contain four rows of Ge rods in air represent high coupling of incident Gaussian waves to the outputs of waveguides under $\eta_p = 20\%$ (Fig. S2(a–c)); also, show high interaction of the guided modes to the two rows which surrounded the waveguides. Owning to the position disordering, the rods in different positions induce a phase difference which results in constructive and destructive interferences in the transmitted light waves; therefore the H-field distributions have been monitored at different normalized frequencies $\frac{a}{\lambda} = 0.435, 0.433$, and 0.4 for straight, L-shaped, and crossing waveguides. As obvious from the H-filed distribution under a position disordering $\eta_p = 20\%$, the sharp bend of

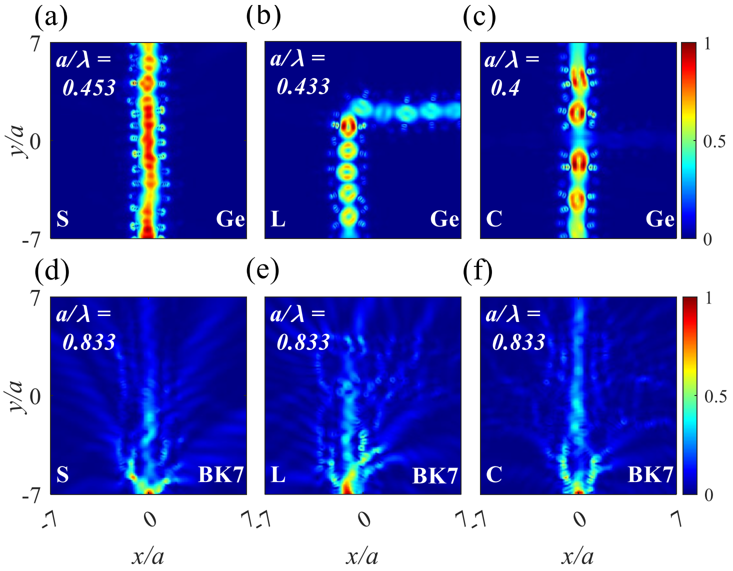

Fig. S2. (a)–(c) H-field distributions of Ge-based MMs of B-type straight (S), L-shaped (L), and crossing (C) waveguides at $\frac{a}{\lambda} = 0.453, 0.433$, and 0.4, respectively under $\eta_p = 20\%$. (d)–(f) H-field distributions of BK7-based PCs of B-type of S, L, and C waveguides, respectively, under $\eta_p = 20\%$ at $\frac{a}{\lambda} = 0.833$.

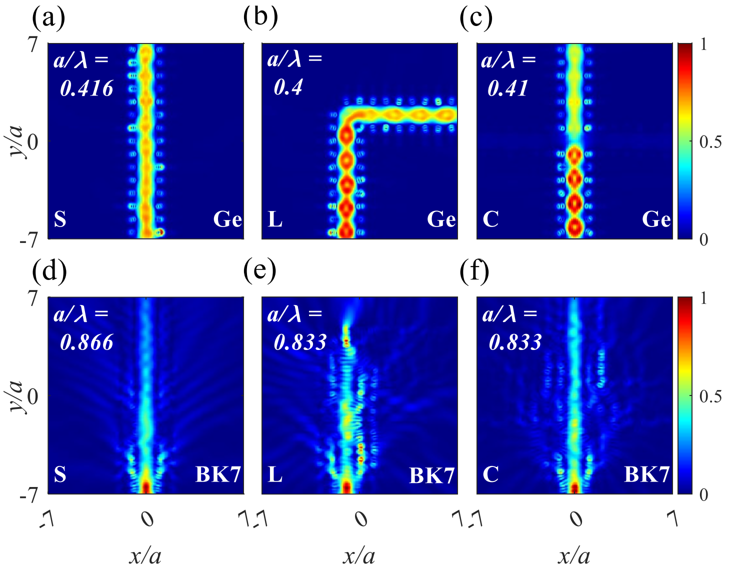

Fig. S3. (a)–(c) H-field distributions of Ge-based MMs of B-type straight (S), L-shaped (L), and crossing (C) waveguides at $\frac{a}{\lambda} = 0.416, 0.4$, and 0.41, respectively under $\eta_r = 13\%$. (d)–(f) H-field distributions of BK7-based PCs of B-type of S, L, and C waveguides, respectively, under $\eta_r = 13\%$ at $\frac{a}{\lambda} = 0.833$.

L-shape waveguide and a horizontal line defect in the crossing waveguides do not affect the guided modes. H-field distributions of the B-type BK7 PCs of straight, L-shaped, and crossing waveguides at $\eta_p = 20\%$ show penetration of incident light waves over the whole structure (Fig. S2(d–f)). The sharp bend in the L-shaped waveguide and the horizontal line defect in the crossing waveguides affect strongly the guided modes.

H-field distribution for the B-type straight, L-shaped, and crossing waveguides under radius disordering $\eta_r = 13\%$ show the high coupling of incident light waves to the outputs for Ge MMs (Fig. S3(a–c)) and very low coupling of incident light to the outputs for the BK7 PCs (Fig. S3(d–f)). For the Ge-based waveguides under radius disordering the sharp bend and horizontal line defects do not influence the guided modes while for the BK7-based wavaeguides the sharp bend and horizontal defects change the guided modes to radiation modes.

TM and TE Mie bandgaps in Te, Ge, and Si metamaterials

By decreasing the refractive index of elements in 2D all-dielectric MMs from Te ($n_o = 4.8$ and $n_e = 6.2$) to Ge ($n = 4$) and Si ($n = 3.48$), the TE_{01} Mie bandgap mode vanishes. In comparison, by decreasing the refractive index of the elements, the TM Mie bandgaps remain which also tolerate disordering. The MMs contain 15×15 Te rods in air under illumination of TE polarized waves show a weak toleration of TE_{01} to rod-radius disordering of $\eta_r = 20\%$ (Fig. S4(a)). Also, the structure under TM polarized waves reveal TM_{01}, TM_{11}, TM_{21}, TM_{02}, and TM_{12} over $0.12 < \frac{a}{\lambda} < 0.18$, $0.23 < \frac{a}{\lambda} < 0.31$, $0.35 < \frac{a}{\lambda} < 0.43$, $0.48 < \frac{a}{\lambda} < 0.56$, and $0.6 < \frac{a}{\lambda} < 0.67$, respectively, in which TM_{01} tolerates rod-radius robustness of $\eta_r = 34\%$ with the inevitable cost of the bandwidth narrowing (Fib. S4(b)). Owning to the rod-radius disordering, the radius of each rod changes, resulting in changes in the localized modes; the changes degrade the robustness to rod-radius disordering, whereas the TM_{01} Mie bandgap shows high robustness due to the supporting of the TM_{01} localization mode for a large variety of radii. As

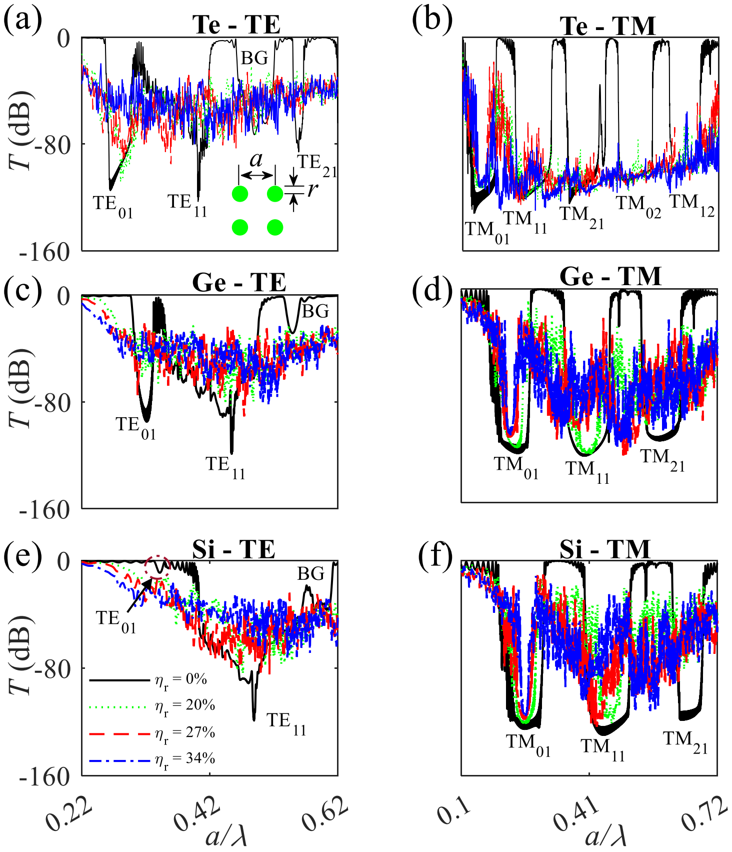

Fig. S4. Logarithmic transmission spectra of Te MMs contain 15×15 Te rods in air under illumination of TE (a) and TM (b) plane waves, respectively. The spectra of 15×15 Ge rods in air under illumination of TE (c) and TM (d) plane waves, respectively. The transmission spectra of 15×15 Si rods in air under illumination of TE (e) and TM (f) plane waves. Solid black, dotted green, dashed red, and dot-dashed blue curves represent the radius disordering of $\eta_r = 0, 20, 27$ and, 34%, respectively.

rod-radius disordering is increased, the quasi-bond states between rods are weaken, which results in escaping electromagnetic waves between the rods and disappearing bandagps' edges.

A Ge-based MMs contain 15×15 elements under TE polarization incident plane waves at $0.22 < \frac{a}{\lambda} < 0.62$ reveal a weak and narrow TE_{01} bandgap at $0.3 < \frac{a}{\lambda} < 0.33$, and the narrow TE_{11} and weak Bragg bandgaps (BG) that do not show robustness to radius disordering (Fig. S4(c)). In contrast, the structure under illumination of TM plane waves over $0.1 < \frac{a}{\lambda} < 0.72$ demonstrates three intense-broad TM Mie bandgaps of TM_{01} ($0.17 < \frac{a}{\lambda} < 0.266$), TM_{11} ($0.351 < \frac{a}{\lambda} < 0.461$), and TM_{21} ($0.53 < \frac{a}{\lambda} < 0.63$). TM_{01} show rod-position robustness of $\eta_r = 34\%$ with inevitable cost of band narrowing, TM_{11} represents robustness of $\eta_r = 20\%$, and TM_{21} does not show robustness to radius disordering (Fig. S4(d)).

In Si-based MMs, TE_{01} approximately disappeared (Fig. S4(e)). In contrast, the structure reveals the strong and broad Mie bandgaps of TM_{01} ($0.21 < \frac{a}{\lambda} < 0.3$), TM_{11} ($0.4 < \frac{a}{\lambda} < 0.5$), and TM_{21} ($0.62 < \frac{a}{\lambda} < 0.68$), respectively, in which TM_{01} shows a strong robustness to radius disordering of $\eta_r = 34\%$ (Fig. S4(f)).

Material dispersion of Te, Ge, Si, and BK7

Te is an anisotropic material with $n_0 = 4.8$ and $n_e = 6.2$ under TE and TM polarized waves at $4\ \mu m < \lambda < 10\ \mu m$[1]. As obvious from Fig. S5, Ge and Si show approximately constant n = 4 and 3.48, respectively, over a wavelength range of $1 < \lambda < 10$ μm. Also, BK7 reveals n = 1.5 over the wavelength range of $1 < \lambda < 2.5\ \mu$m. As a result, Ge and Si materials can be used over a broad range of $1 < \lambda < 10\ \mu$m. It is worth mentioning that the extinction coefficient for Te, Ge, Si, and BK7 over the wavelength ranges is approximately zero[2–4].

In terms of fabrication, many disordered structures have been fabricated and characterized. In 2019, Milosevic et al. experimentally showed a hyperuniform structure consisting of disordered air holes on a silicon-on-insulator platform. They designed and fabricated active and passive elements of optical modulators and resonators, respectively, that utilize disordering[5]. In 2019, Zhou et al. designed and fabricated an ultra-broadband polarizer containing disordered metamaterials based on silicon photonics[6]. In 2020, Wohlweed et al. designed and fabricated Cu-Sn disordered network of metamaterials at the nanoscale. They show that frequency-selective absorption in the disordered metamaterials can be controlled[7]. In 2020, Liu et al. fabricated disordered gyromagnetic photonic crystals, and their results showed that increasing disorders transforms trivial insulators to nontrivial topological insulators[8]. In 2020, Aubry et al. presented a transport experiment of the dielectric cylinder with a refractive index of 6 at the microwave regime. Their results represented that the disordered structure of the dielectric arrays can show transparency, photon diffusion, and a complete bandgap[9].

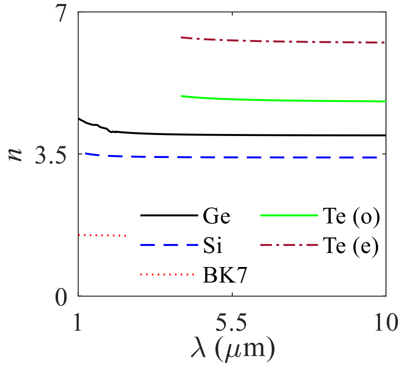

Fig. S5. Material dispersion for Te, Ge, Si, and BK7.

References

1. Caldwell, R. S. & Fan, H. Optical properties of tellurium and selenium. *Phys. Rev.* **114**, 664 (1959).
2. Amotchkina, T., Trubetskov, M., Hahner, D. & Pervak, V. Characterization of e-beam evaporated ge, ybf 3, zns, and laf 3 thin films for laser-oriented coatings. *Appl. Opt.* **59**, A40–A47 (2020).
3. Schott zemax catalog 2017-01-20b, schott glass data sheets. *http://www.schott.com* .
4. Li, H. Refractive index of silicon and germanium and its wavelength and temperature derivatives. *J. Phys. Chem. Ref. Data* **9**, 561–658 (1980).

5. Milošević, M. M. *et al.* Hyperuniform disordered waveguides and devices for near infrared silicon photonics. *Sci. reports* **9**, 1–11 (2019).

6. Zhou, W., Tong, Y., Sun, X. & Tsang, H. K. Ultra-broadband hyperuniform disordered silicon photonic polarizers. *IEEE J. Sel. Top. Quantum Electron.* **26**, 1–9 (2019).

7. Wohlwend, J., Sologubenko, A. S., Dobeli, M., Galinski, H. & Spolenak, R. Chemical engineering of cu–sn disordered network metamaterials. *Nano Lett.* (2021).

8. Liu, G.-G. *et al.* Topological anderson insulator in disordered photonic crystals. *Phys. Rev. Lett.* **125**, 133603 (2020).

9. Aubry, G. J. *et al.* Experimental tuning of transport regimes in hyperuniform disordered photonic materials. *Phys. Rev. Lett.* **125**, 127402 (2020).
